# The relationship between mass customization and sustainable performance: The role of firm size and global E-commerce

**DOI:** 10.1016/j.heliyon.2024.e27726

**Published:** 2024-03-11

**Authors:** Guan Hui, Abdullah Al Mamun, Mohammad Masukujjaman, Zafir Khan Mohamed Makhbul, Mohd Helmi Ali

**Affiliations:** a School of Foreign Languages, Changzhi University, Changzhi City, Shanxi Province, 046000, China; b UKM - Graduate School of Business, Universiti Kebangsaan Malaysia, 43600, UKM Bangi, Malaysia; c Faculty of Business Management and Professional Studies, Management and Science University, 40100, Shah Alam, Selangor, Malaysia

**Keywords:** Competitiveness, Cross-border eCommerce, Manufacturing small-to-medium sized enterprises, Mass customization capability, Sustainable performance

## Abstract

The formation of mass customization competencies is crucial for the increasing number of manufacturing companies in modern times. This study assessed the relationship between mass customization capability and its determinants on sustainable performance. Additionally, it explores the mediating role of mass customization capability and sustainable performance, while also examining the moderating effects of firm size and cross-border eCommerce in these associations. The study used online survey data from 339 manufacturing small-to-medium-sized enterprises in China to test the hypothesized relationships. The collected data were analyzed using partial least square structural equation modeling and necessary condition analysis. The results indicated that flexible manufacturing competencies, modular product architecture, and customer relationship management are significantly and positively connected to mass customization capability. Moreover, the study observed that mass customization capability and competitive pressure have a significant positive influence on the sustainable performance of Chinese manufacturing SMEs. The findings also revealed that firm size and cross-border e-commerce engagement have a negative and positive moderating effect, respectively, between mass customization capability on sustainable performance, which confirms a relatively higher effect of customization capability on sustainable performance among smaller firms and firms engaged in cross-border eCommerce. Fundamentally, these findings can lead to the development of a comprehensive framework to promote mass customization capability, cross-border e-commerce, and sustainable development of manufacturing small-to-medium-sized enterprises China.

## 1 Introduction

Currently, mass customization is becoming a vital issue to enhance the competitiveness of enterprises in the current fast-evolving business landscape [1]. SMEs are putting more effort into getting to know their customers and meeting their unique needs. They are also turning to a mass customization business strategy to meet these needs and attain a competitive edge [2,3]. Mass customization capability (MC) is the capacity to supply modified goods or services that fills the individual needs of each consumer without making significant sacrifices in cost, delivery, or quality [4]. As organizations strive to meet diverse customer needs and preferences, the extent to which they can tailor their products, services, and processes becomes pivotal. Even though mass customization in China has many benefits for businesses, not many companies conduct it on a large scale, particularly small start-ups [5]. However, the determinants that contribute to the development and enhancement of customization capability remain poorly understood. Consequently, there is a pressing need for comprehensive research to elucidate the factors influencing customization capability and to assess their subsequent impact on sustainable performance.

There has been a meteoric rise in the number of scholarly works discussing the factors that make mass customization possible [6]. For instance, Alshura's [7] research underscores the vital link between CRM capabilities and forging a sustainable competitive edge, underscoring the influence of customer relationship management on sustainable performance. Grawe et al. [8] emphasize the connection between service innovation and market performance, signaling the importance of innovation capabilities in steering sustainable outcomes. Chatterjee et al. [9] delves into absorptive capacity and dynamic capability's impact on ubiquitous CRM adoption, spotlighting their role in operational sustainability. Piller & Blazek's [10] work zeroes in on sustainable mass customization's core capabilities, illuminating the specific skills necessary for sustainable customization practices. Likewise, Hassan et al. [11] establishes a positive, significant correlation between customer integration and sustainable performance, reinforcing the impact of customer-centric factors on sustainability outcomes. Particularly, even though research on the factors that affect MC has proliferated in the last 20 years [6,12,13], scholars posited that relevant study on MC is still immature and needs to be further explored [14]. Those existing literature offers limited insights into the specific drivers that propel customization capability within organizations, hindering the development of targeted strategies for customization enhancement. Moreover, the relationship between customization capability and sustainable performance remains ambiguous, with insufficient empirical evidence to validate the purported benefits. None of these studies focuses on incorporating external strategies, such as competitive pressure, as a variable to investigate its diverse effects on sustainable performance (SP). Additional research is warranted to bridge the gaps in the literature on mass customization (MC) and sustainable performance (SP).

Recently, there has been a noteworthy development in the field of cross-border e-commerce (CBEC), which has developed as a new avenue for economic and business cooperation between nations [15]. CBEC is a commercial model in which goods are sold directly to foreign customers via retailers or markets using online channels [16]. In 2018, business-to-consumer CBEC transactions were worth more than US$650 billion worldwide, and 52.2% of all online shoppers in the world used CBEC [17]. CBEC is an integral part of business innovation that can help improve job performance [18]. However, there is scant research on how CBEC affects the economy and the environment to speed up the long-term improvement of Chinese businesses, specifically small and medium-sized businesses. Even fewer are those studies conducted to investigate CBEC as a firm strategy to enhance mass customization and measure enterprises’ long-term performance. Hence, this research seeks to fill these deficiencies by developing a framework based on MC.

There are differences between SME businesses, just as there are differences between SMEs and large businesses. Russo and Tencati [19] studied corporate social responsibility practices and found that SME businesses all had different ways of managing and acting responsibly. As a consequence of this significant finding, they called for more research to probe how small and medium-sized businesses differ in how they handle common management issues like sustainability. However, thus far, almost all research on the differences in firm size has been done on SME firms versus large firms [20]. We did not find any studies addressing this critical emerging research need that Russo and Tencati [19] indicated from the perspective of MC and measuring SP. Moreover, existing research, such as Deshpande [21], has explored the relationship between mass customization and financial and market performance. Similarly, Liu et al. [22] and Trentin et al. [14] have identified the connection between mass customization and business performance. The study by Sheng et al. [1] investigated the correlations among mass customization, environmental performance, operational performance, market performance, and economic performance. However, it is important to note that the concept of sustainable performance has not been addressed as a distinct factor previously. Taking this aspect into account could contribute to a more comprehensive understanding of the relationship between mass customization capability and sustainable practices.

The arguments above show a deficiency in the literature regarding our understanding of how to measure sustainability performance in the context of mass customization, how CBEC helps sustainability development, and how the sustainability performance of SME firms is different. Thus, the present study aims to diagnose the connection between MC, and its determinants, on SP. The study also investigates the mediating relationship of MC and SP along with moderating effects of firm size and CBEC within the same relationships. The present study contributes to the literature on Customer Relationship Management (CM) by proposing a new framework with MC enablers and firm external strategy (pressure of competition) in the path of SP enhancement in SMEs in China. With its advanced analysis (necessary condition analysis), this study offers various novel insights for managers to manage MC and understand how firm performance is affected by cross-border e-commerce ensuring SP within SMEs.

The remainder of this paper is structured as follows. Section 2 reviews the relevant literature and forms the hypotheses. Next, Section 3 presents the research methodologies, followed by the result of the study. Section 4 discusses the results, while Section 5 elucidates the study's implications. Finally, Section 6 concludes the paper by providing the study's limitations and directions for future research.

## 2 Literature review

### 2.1 Theoretical underpinning

#### 2.1.1 The resource-based view (RBV)

RBV offers a theoretical basis for studying these links in more depth. The RBV suggests that resources and skills that lead to a lasting competitive advantage are beneficial to customers and unique and impossible for competitors to copy [23]. The RBV theory suggests that a firm's competitive edge in the market arises from its distinct competence. This concept aids the firm in systematically blending internal resources with competitive advantages, facilitating the resolution of associated issues [24,25]. Teece's exploration of the RBV model validates that a firm's resources refer to the tangible stock it presently controls, while dynamic competencies pertain to its skill in effectively leveraging these assets to achieve desired outcomes [26]. Furthermore, Xie et al. [27] propose that a firm's unique resources and its proficient utilization thereof play crucial roles in establishing a competitive advantage. According to Muangmee et al. [24], a company's strategies are intricately linked to the resource utilization and execution of dynamic capabilities. Notably, the implementation of dynamic competencies aligns with a firm's pursuit of green innovation.

Relating to our current study, resources and skills like technical knowledge and ability and a multi-talented workforce add value to the firm and help it turn a short-term competitive advantage into a long-term one [21,28]. In addition, MC has all of the same qualities as RBV. MC is critical to organizations because it helps them meet customer needs. From a strategic point of view, MC helps companies provide customers with the right amount of product at the right price and time [29]. Businesses that do not have MC can fail to offer the appropriate level of customization in a reasonable amount of time with consistent quality.

### 2.2 Mass customization capability, sustainable performance and its relationships

The term “MC” was initially used in a study by Tu et al. [30]. MC is defined as the ability to produce, distribute, and deliver a wide variety of customer-specific goods and services, while also managing costs by offering more standardized goods and services [30]. Studies have examined different ways to get MC, and the scope of MC literature has grown over the years to include technological-level, individual-level, and organizational-level enablers, such as modularity [31], absorptive capacity [29], internal or external learning and effective processes implementation [32], customization knowledge utilization, and business processes. These studies concluded that companies should focus on putting in place practices that could result in greater flexibility and responsiveness if they want a mass customization system to work.

The present study discusses mass customization enablers in a context that promotes the MC of manufacturing SEMs in China. Technical knowledge and abilities [33] from technological-level enablers, flexible manufacturing competencies, modular product architecture [34] from organization-level enablers, collaborative relationships [35], multi-talented workforce [36], customer relationship management [2] from individual-level enablers were selected as variables to measure their impact on mass customization.

SP is when ecological, monetary, and social performance are all considered. This is good for the environment and society and provides businesses with a competitive edge as well as economic sustainability benefits [37,38]. Economically SP happens when a company grows its market portfolio and strengthens its status in the market while making money and getting a return on investment [39]. The environmental SP shows how a company's sustainability activities positively affect the natural environment, inside and outside the company [40]. The environmentally friendly output includes a decrease in CO2 emissions, wastewater, solid waste, energy use, dangerous synthetic compounds, and material use, as well as a stronger adherence to environmental standards and well-designed goods and packaging that can be reused, fixed, or recycled [41]. In this article, we investigate environmental and economic SP.

The relationship between mass customization capability and sustainable performance is synergistic. When harnessed effectively, mass customization can contribute to resource efficiency, waste reduction, and localized production, all of which are key components of sustainable practices. Mass customization capability can contribute to sustainable performance by optimizing the use of resources. Businesses with mass customization capabilities can produce goods and services based on actual demand, reducing overproduction and minimizing waste [42]. This aligns with sustainable practices that aim to conserve resources and minimize environmental impact [14]. As Piller and Müller [43] stated that businesses with mass customization capabilities can maintain lower inventory levels by producing products on demand. This can reduce the risk of unsold inventory and the need for disposal, aligning with sustainability goals of minimizing waste. Likewise, according to Sheng et al. [1] developing a strong mass customization capability can differentiate a business in the market and provide a competitive advantage. Integrating sustainable practices into this capability can enhance the business's reputation and appeal to environmentally conscious consumers. This enhances the sustainable performances. As per Dissanayake [44], mass customization often involves closer interaction with customers, which provides opportunities for education about sustainable choices and practices. Businesses can use this engagement to raise awareness about eco-friendly options and encourage customers to make environmentally conscious decisions.

### 2.3 Cross-border electronic commerce

The term “cross-border electronic commerce” (CBEC for short) is used to describe the practice of selling goods to customers in a different country via the internet. This can be done either directly via a company's website (i.e., “business-to-consumer” or “B2C”) or indirectly via a third party (i.e., “business-to-business-to-consumer” or “B2B2C”) [16]. Cross-border e-commerce is now a key way to promote international business [15] as it offers developed and developing nations the chance to benefit from international transactions [45,46]. In 2018, 51.2% of all people who bought things online did so across international borders [47]. In 2018, worldwide B2C cross-border e-commerce reached $676 billion, a 27.5 percent increase from the previous year. It is believed that this amount will be more than $800 billion in 2019 [47]. What needs to be determined from the existing literature is whether or not the growth of CBEC can help Chinese businesses grow sustainably.

### 2.4 Hypotheses development

#### 2.4.1 Enablers of MC

A collaborative relationship (CR) with customers provides companies with a great deal of knowledge on the basis that they can innovate new product designs, establish solution space, improve the existing product and component designs, change process sequences of new production, and adjust existing ones [29]. The companies must also work with their suppliers to get their combined knowledge and resources. In addition, they need to respond swiftly and accurately to the needs of the final client by coordinating the various tasks involved in product design creation, improvement of process, and delivery of products [35,48,49]. For manufacturing SMEs, this can mean sharing insights on production processes, market trends, and customer preferences. Access to such information can enhance the firm's understanding of customization needs and improve its ability to tailor products accordingly. Collaborative relationships positively affect the Mass Customization capabilities of manufacturing SMEs by facilitating resource pooling, supply chain integration, agile responses to market changes, co-innovation opportunities, customer-centric focus, risk mitigation, and access to new markets. These factors collectively contribute to the enhancement of SMEs' ability to deliver customized products efficiently and effectively. Hence, we formulate the following hypothesis:

Hypothesis (H1a) CR positively affects the MC of manufacturing SMEs.

Managers need the right information at the right time to make the right decisions [50]. ICTs, which stands for “Information and communication technologies,” offer ways to connect people inside and outside a country [21,49]. ICT is a driving force behind mass customization because they are quite flexible, efficient, and quick to respond [51]. Bhatt et al. [52] examined real-world data and concluded that the flexibility of an organization's information and communication technology infrastructure makes it more competitive and responsive. Mass Customizers must pick and apply pertinent information and communication technologies tools to collect and analyze large amounts of data relating to customer order-specific requirements, accessibility of materials and parts, delivery period, and the status of delivery, among others [33,53]. Deshpande [21] found that knowledge scanning, meaning the capture of internal and external information, and technology and its current status significantly affect MC or an organization. Manufacturing SMEs with technical expertise are better positioned to troubleshoot issues, adapt to unexpected circumstances, and maintain a smooth flow in their customization operations. Technical proficiency enables innovation, optimization of customization processes, seamless technology integration, adaptability to evolving technologies, quality assurance, efficient collaboration with technology providers, facilitation of continuous improvement, and enhanced problem-solving abilities. Hence, we suggest the following hypothesis:

Hypothesis (H1b) Technical knowledge and abilities (TK) positively influence the MC of manufacturing SMEs.

A manufacturing company's capacity flexibility is its ability to change its available capacity in a way that is easy and cheap to meet the needs of the moment. Ullah and Narain [53] indicated that flexible manufacturing (FM) capability is an organization's ability to deliver a diverse range of product varieties efficiently based on cost, quality, and time. FM is a group of internal skills needed for an organization to be competitive. These skills include flexibility in the machine, labor, managing materials, and routing [2]. Tseng and Radke [54] argued that being good at flexible manufacturing reduces the costs of making custom products. This can be made easier by using constant improvement programs, time-specifying manufacturing, and integrating processes and functions. Existing research shows that MC requires different strategies and practices to increase the flexibility of production so that changes in what customers want do not cause problems with the business [6,55]. Salvador et al. [56], reported that a manufacturer's MC is affected by the advancement of flexible manufacturing resources, which include resources; tangible and intangible. These resources make it possible to make and deliver products in different types and quantities. Recently, Ullah and Narain [2] revealed that flexible manufacturing competence has significantly influenced the MC in the Indian context. Thus, we suggest the following hypothesis:

Hypothesis (H1c) FM positively influences the MC of manufacturing SMEs.

Modular product (MP) design offers customers a wider range of options and makes the manufacturing system much more flexible [57]. The company can simultaneously get economies of scale and scope as MP [6]. According to scholars [34,58], MP helps reduce the time to develop and deliver a product. In addition, it reduces the number of components, increases productivity, lowers inventory levels, and diminishes the risk of having obsolete inventory. It also improves the design of the process, making it easier to implement mass customize [57]. Modular architecture also makes it possible to change the design of a product based on what has been learned about customization in the past [59]. Customers want personalized products and faster shipping simultaneously. This means modular product architecture must be used [33]. According to researchers [34,59], MP is a design capability that is significantly connected with mass customization implementation. Therefore, Modular Product design positively influences the Mass Customization (MC) capabilities of manufacturing SMEs by offering flexible configuration, enabling rapid customization, supporting scalability and variety, promoting cost-efficiency, reducing time-to-market, improving quality control, involving customers in the design process, adapting to changing trends, and enhancing supply chain coordination. This approach empowers SMEs to efficiently deliver customized products while maintaining operational effectiveness. Our hypothesis is postulated as follows:

Hypothesis (H1d) MP positively influences the MC of manufacturing SMEs.

For companies to reach their goals, they need a team of people with different skills working together. If the company holds employees whose attributes are cross-training, high skill, and who can apply their skills to get better results, the company can reach an extent of performance that would not have been possible without them [28,36,60]. Ketkar and Sett [61] examined how changes in the environment affect the performance of an organization and how the flexibility of the human resource system plays a role. These workers help the company use time-based manufacturing practices, like fixing problems on the shop floor, redesigning equipment, and working to improve quality [62]. Jiang et al. [28] conducted research that proposed the integration of suitable methods, such as collaborative problem-solving, offering incentives for demonstrating learning behaviors, and involving employees in decision-making processes. These approaches are recommended to stimulate the cultivation of tacit knowledge within the organization and to guarantee favorable results for the organization as a whole. Ullah and Narain [63] emphasized that nurturing favorable employee conduct through provisions such as training, education, job rotation, enlargement, enrichment, and empowerment can equip companies can not only address and adapt to shifts in market demand but can also position themselves to excel in a business environment that increasingly values customization. In summary, a skilled, adaptable, and empowered workforce is key to navigating the complexities of meeting individual customer needs at scale, which aligns with the core principles of mass customization. The following hypothesis is proposed:

Hypothesis (H1e) Multi-talented workforce (MT) positively influences the MC of manufacturing SMEs.

For mass customization to work, it is clear that the customer must be involved in the process of creating value. Owing to this, it is vital to keep a better relationship with customers. The main purpose of CM is to aid in explaining customers' needs to ensure that these requirements can be met fast, accurately, and efficiently [64]. Once clients are perfectly satisfied, businesses can hope that they will buy from them again and keep them as customers. CM also lets companies tell the difference between low-value and high-value customers so they can offer each one the right level of service [65]. With the assistance of customer management, companies can keep track of their customer's expectations, behaviors, and buying patterns and build expertise that can be used to make plans for the future. CM positively influence the MC of SMEs through mechanisms such as enabling SMEs to gather and analyze customer data, preferences, and feedback for accurate product tailoring. The insights gained from CM aid in creating personalized offerings aligned with individual customer requirements, enhancing value proposition and satisfaction. Improved communication facilitated by CM allows SMEs to gather real-time feedback, address concerns, and create a collaborative, customer-centric environment. Integrating CM with product design platforms enables direct customer involvement, ensuring the final customized output aligns closely with customer expectations. By giving employees flexible workplace rules and less formalization, a company that encourages creative, independent, self-directed learning and working would offer something unique to its customers [66]. Recent studies, such as those by Ullah and Narain [2,63], found a significant correlation between CM and MC. Therefore, we propose the hypothesis as follows:

Hypothesis (H1f) CM positively influences the MC of manufacturing SMEs.

#### 2.4.2 The mediating role of MC

Customizing products is the best way to meet customers’ needs and consider their different cultures, but it will only continue if it can make enough money to keep going. Hence, choosing to customize a product will only make economic sense if the return is higher than the return that the company can get from other investments. This is called the opportunity cost [67]. Companies must have benefits in their portfolios that help mark up sales volume to keep generating revenues. The effect on portfolio sales could be considered an extra sale that makes it possible to sell more products. Companies can change the standards for their products to open up more new markets and offer themselves extra competitive advantages [68]. The mass customization ability influences the sustainable performance through a reduction in waste and resource optimization, a precise inventory management and customized production practices that minimize environmental impact. Meeting diverse customer needs through mass customization enhances customer satisfaction, fostering loyalty and reducing the reliance on excessive marketing efforts. The flexibility and adaptability of SMEs equipped with mass customization capabilities enable them to respond quickly to market shifts, minimizing obsolete inventory and advancing a more sustainable business model.

Mass customization acts as a mediator connecting enablers with sustainable performance for SMEs. Implementing it effectively allows SMEs to harness these enablers better, enhancing sustainable outcomes. Collaborative relationships improve information sharing and supply chains [48]. Technical expertise is vital for mass customization, including advanced tech and quality control [21]. Quick adaptation via flexible manufacturing is crucial, meeting changing demands [2]. Modular designs aid customization [59], combining efficient production with tailored solutions. A diverse workforce supports various customization aspects. Effective CM grasps customer needs, vital for tailored products [2]. Therefore, strong enablers empower SMEs' customization, enhancing sustainable outcomes via waste reduction, satisfaction, and resource efficiency. Thus, this study will test the mediating role, and the hypothesis is formulated as follows:

Hypothesis(H2a) MC plays the positive mediating role between MC enablers and SP of manufacturing SMEs.

#### 2.4.3 Competitive pressure

Competitive pressure (CP) research in China has been trending for a while now. In the past few years, research on competitive pressure has spread to many different fields, such as government spending on science and environmental control [69], technology invention and reducing pollution in the industrial sector [18,70]. In addition, substantial literature [[71], [72], [73], [74]] uses businesses as the focus of research having relationship with the business performances. Moreover, competitive pressure affects how much debt companies have and how they use their resources [75]. Competitive pressure encourages innovation, efficiency, and sustainability in manufacturing SMEs, fostering the development of environmentally and socially responsible practices and technologies. This responsiveness to market demands aligns SMEs with changing consumer preferences, enhancing their competitiveness and long-term viability. Additionally, collaboration, regulatory compliance, and a positive brand image further contribute to the overall sustainability and resilience of these businesses in the face of competition. Therefore, the following hypothesis is postulated:

Hypothesis(H2b) CP positively influences the SP of manufacturing SMEs.

#### 2.4.4 The moderating role of firm size and cross-border eCommerce

Different business strategies, like how they hire people, are affected by the size of the company (i.e., smaller companies usually hire people they know or have worked with before, while bigger businesses hire people based on their skills). Due to this, they use marketing and management techniques in different ways [76]. These differences are essential in terms of sustainability and might lead to different performance outcomes.

The company size affects how well the organization does. For example, there is evidence that bigger companies tend to focus more on marketing than smaller ones [77]. Large firms possess ample resources, enabling them to effectively translate their marketing expertise into valuable performance measures. Large firms possess costly technological and information systems due to their superior ability to benefit from economies of scale compared to smaller firms [78]. However, this advantage also can restrict their capacity to manufacture customized goods. Large firms may encounter difficulties in managing workers, particularly in terms of exerting management control, which can negatively impact the company's performance [77,79]. These problems point to poor internal coordination, one of the main reasons sustainable projects fail [80].

Therefore, we predict that the relationship between mass customization capability and sustainable performance can vary based on firm size. Large firms may possess intrinsic benefits in terms of resources and economies of scale, which could potentially undermine their ability to adapt to mass customization and maintain sustainability. Smaller companies can benefit from targeting specific customization markets and being quick to respond to sustainability trends. This can enhance the impact of their customization abilities and commitment to sustainability. Therefore, we propose the following hypotheses:

Hypothesis(H3a) FS has a negative moderating role in MC- SP relationships.

Most research on CBEC has examined how the success of CBEC is affected by factors from the buyers' point of view [81]. Watson et al. [82] studied from the point of view of customers and sellers. For example, to predict the likelihood of a sale in CBEC, a theoretical model of sellers' trust in buyers has been put forward [83,84]. Compared to traditional e-commerce, CBEC research usually looks at the achievement of CBEC channels from the sellers’ point of view [85]. Last, some researchers state that CBEC needs new models and theories, which is also a trend in this field [85,86].

Most of the time, it was noticed that scholars have examined CBEC in the context of buyers and sellers. CBEC acts as a catalyst that amplifies the positive impact of mass customization capability on sustainable performance. The alignment of these two factors allows companies to expand their global reach, leverage customer insights, develop agile supply chains, foster customer engagement, optimize resources, drive innovation, and manage risks effectively. This harmonious relationship creates a synergistic effect that not only enhances customization efforts but also contributes to sustained and thriving business performance in a global context. The relationship between mass customization capability and sustainable performance can be positively influenced by increased cross-border e-commerce engagement. The exposure to a global market encourages businesses to invest in customization technologies and sustainable practices to remain competitive and meet diverse consumer expectations. On the other hand, decreased cross-border engagement may weaken both customization capabilities and sustainability efforts as businesses may prioritize more standardized approaches for a narrower market. Thus, we suggest the following hypothesis:

Hypothesis(H3b) CBEC has a positive moderating role in MC-SP relationships.

## 3 Research methodology

**Fig. 1:**
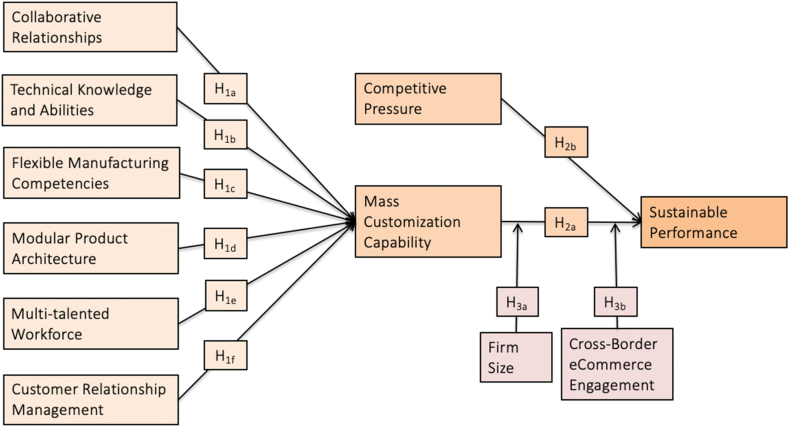
Research framework.

This research used a deductive and quantitative approach to explore the effect of MC and SP, as presented in Fig. 1. The data were gathered in a cross-sectional research design. Specifically, data were collected once from each respondent, unlike the longitudinal research. We used the PLS-SEM method of causal-predictive data analysis to test the hypotheses.

### 3.1 Sample and population

The target populations are the top and mid-level management staff of manufacturing SMEs in China, where the unit of analysis is manufacturing SMEs. Using the G-power 3.1 tool with 10 different predictors, a power of 0.95 and an effect size of 0.15 were utilized to estimate the sample size. The least samples needed to analyze with enough power was 145 [87]. Still, Hair et al. [88] recommend using at least 200 samples in PLS-SEM. However, to avoid the restrictions of a low sample size, this research intended to obtain data from more than 300 top and mid-level management staff from manufacturing SMEs in China.

### 3.2 Survey instrument

The survey questionnaire was used for this research designed by applying simple and neutral wording to allow the participants to understand the questions easily. To capture collaborative relationships, 4 question items were adopted from Jitpaiboon et al. [35], four items from Peng et al. [33], for technical knowledge and abilities, and four items for flexible manufacturing competencies from Ullah and Narain [2]. Referring to Zhang et al. [34], modular product architecture was measured using four items. Three items were retrieved from Huang et al. [32], to assess the multi-talented workforce, whereas four items were modified from Ullah and Narain [2] to examine customer relationship management. While four items were borrowed from Huang et al. [32], to measure the mediator, and MC. Four items were borrowed from Seigyoung's [89] test for the moderator, competitive pressure. We also adopted 5 items from Yusliza et al. [90] to measure the company's SP. The questionnaire was based on the ideas we discussed so far, and we used a 5-point Likert scale for each question ranging from 1 (significantly disagree) to 5 (significantly agree). Completed questionnaire submitted as (Supplementary Material S1. Survey instrument).

### 3.3 Data collection

Shaanxi, Zhejiang, and Guangdong are the three provinces that we have decided to use as our examples of representative areas as they are key manufacturing bases. Among them, Guangdong, a South Chinese province, shows a higher level of digital transformation and upgrading, more robust product customization capabilities, and a higher volume of CBEC sales of manufacturing factories. Shaanxi, located in northwest China, is an old industrial province with a low ability to customize products. CBEC is also not used much in manufacturing factories to increase business turnover. Zhejiang, located in Eastern China, shows an evolving industrial base, a resilient mass customization development, and an explosive cross-border eCommerce acceleration. These three regions signify different phases and stages of mass customization capabilities and cross-border eCommerce engagement [91].

The study uses a back-translation technique to make sure the survey was accurate. At first, the research questionnaires were carefully looked through using English literature [92]. Second, we took help from professional experts who are used to doing research and spoke English and Chinese to translate it into Chinese. Third, two professional translators who spoke English and Chinese did a blind translation of the Chinese questionnaire back into English. Fourth, the quality of the translation was judged by contrasting the two versions. When there was dispersion, researchers and translators worked to find a solution. Last, the questionnaire was pretested to see how accurate it was. The set of questions was then changed and confirmed.

We undertook a pilot test to better understand the survey contexts. The issuance and completion of questionnaires were closely supervised to ascertain the validity and well-organized data gathering. We sent 32 questionnaires and collected them from the pretest sample. Pretest findings confirmed the preliminary validity and reliability of the items used [93]. To ensure the correctness and extensiveness of responses, we picked sampled enterprises based on recommendations from the local “Bureau of Commerce” and collected the contact details of randomly selected 431 manufacturing SMEs from Guangdong, Shaanxi, and Zhejiang.

This study uses online surveys as most of the country is under COVID-19 lockdown during the data collection period. Before sending the questionnaires by e-mail, the target enterprises were called on the phone to find the best representative, who then agreed to take part. The respondents were executive, senior, or middle management of the manufacturing SME with sufficient knowledge about the SME. We collected data applying the online survey form (Link: https://www.wjx.cn/vm/hn0jK1e.aspx) shared among the executive or senior management or middle management of the selected manufacturing SMEs from September 2020 to October 2020. We included a few screening questions in the questionnaire to confirm that the data was collected only from the target respondents. This study also obtained participants’ written informed consent to participate before the data collection. A total of 431 executives, senior management, or middle management of the selected manufacturing SMEs were invited to contribute to this survey. There were 339 valid replies to this study, with a useable response rate of 78.7%.

### 3.4 Common method bias

To determine the impact of common method bias (CMB) and come up with ways to fix it, we carefully made the questions and told the people who answered them that there were no absolute right or wrong responses and that their answers would be assessed anonymously [94]. Harman's single-factor test was used as a diagnostic tool for this study to determine the influence of common method bias. The single factor made up 43.87%, less than the prescribed limit of 50% in Harman's one-factor test. This proves that CMB had no significant effect on this research [94]. Additionally, we examined CMB by assessing the full collinearity of all constructs, as suggested by Kock [95]. All of the study's structures regressed on the common variance and the VIF value shown in Table 1. There is no bias in the data coming from a single source as all VIF values are lower than 3.3.

**Table 1:** Full collinearity test.

	CR	TK	FM	MP	MT	CM	MC	CP	SP
Variance Inflation Factors	2.450	2.779	2.634	1.911	2.762	2.609	1.888	1.710	2.424

**Note:** CR: Collaborative Relationships, TK: Technical Knowledge and Abilities, FM: Flexible Manufacturing Competencies, MP: Modular Product Architecture, MT: Multi-talented Workforce, CM: Customer Relationship Management, MC: Mass Customization Capability, CP: Competitive Pressure, SP: Sustainable Performance.

### 3.5 Multivariate normality

This study used Web Power to examine multivariate normality. The skewness, coefficient of kurtosis and p-values for Mardia were all determined by using this tool (Source: https://webpower.psychstat.org/wiki/tools/index). The results of the multivariate normality test demonstrate that Mardia's multivariate skewness and kurtosis have p-values that are less than 0.05 [96]. This proves that Mardia's data is not normal.

### 3.6 Data analysis method

The PLS-SEM was utilized in this investigation because the dataset exhibited multivariate non-normality. This was the reason for its utilization. Hair et al. [97] stated that variance-based SEM could be used to examine data that does not fit the normal distribution. In this study, there were two steps to the PLS-SEM analysis. At first, it estimates the model and evaluates its structural validity and reliability [88]. During the second phase, associations are examined [97].

NCA is a tool for analyzing data that was made to go along with more traditional methods like multiple regression and SEM [98]. The benefit that NCA adds to the analysis of data is a more nuanced knowledge of the conditions necessary for assessment. These requirements variate with types and levels of requirements [98,99]. Additionally, NCA identifies the must-have or bottleneck components required for the result. Dul [100], recommended that the two-stage NCA could be used. In the first step, the effect size and ceiling line of each input variable is calculated to set up the right conditions for the outcome measure [100]. In the second step, the significance level of every input variable's effect size was calculated. The bottleneck analysis was done to see how the various percentile levels of outcome measures were affected by the necessary conditions [98].

## 4 Findings

### 4.1 Demographic characteristics

Referring to Table 2, among the 339 study respondents, 184 (54.3%) are executive or senior management, and 155 (47.5%) are middle management of companies. Male respondents (66.4%) exceeded their female counterparts (33.6%). The age range of the respondents was from 18 to 25 years old (2.1%), 26–35 years old (21.5%), 36–45 years old (53.4%), 46–55 years old (18.6%), and 56–65 years old (4.4%). Out of the 339 people who answered, 62.8% had a bachelor's degree or something similar, 11.8% had a master's degree or something similar, 4.4% had a doctoral degree, and 24.2% had something else. In terms of tenure, most people have worked in manufacturing for 10–15 years, while 15.9% of respondents have been in the industry for more than 20 years, and 17.4% of respondents have worked between 15 and 20 years. Accordingly, 19.5% have been working in factories for 6–10 years, 14.7% for 1–5 years, and 0.4% for less than a year. Among the study respondents, 9.1% worked in clothing and textiles manufacturing, 17.1% were employed in Petroleum, chemicals and plastics, 25.7% were occupied in electronics, computers, and transportation, 10.3% and 4.1 % worked in food production and wood, leather and paper industry, respectively. Last, 33.6 % got a job in metal manufacturing.

**Table 2:** Demographic characteristics.

	N	%		N	%
*Gender*			*Position*		
Female	114	33.6	Executive or senior management	184	54.3
Male	225	66.4	Middle management	155	45.7
Total	339	100.0	Total	339	100
*Age*			*Type of Firm*		
18-25	7	2.1	Clothing and textiles manufacturing	31	9.1
26-35	73	21.5	Petroleum, chemicals and plastics	58	17.1
36-45	181	53.4	Electronics, computers and transportation	87	25.7
46-55	63	18.6	Food production	35	10.3
56-65	15	4.4	Metal manufacturing	114	33.6
Total	339	100	Wood, leather and paper	14	4.1
			Total	339	100.0
*Education*					
Bachelors or equivalent	213	62.8	*Firm Size*		
Master Degree	40	11.8	Small Enterprise	215	63.4
PhD or DBA	4	1.2	Medium Enterprise	124	36.6
Other	82	24.2	Total	339	100
Total	339	100			
			*Cross-Border eCommerce Engagement*		
*Tenure*			None	118	34.8
Less than 1 year	2	0.6	≤20%	101	29.8
1–5 years	50	14.7	21%–30%	24	7.1
6–10 years	66	19.5	31%–40%	23	6.8
11–15 years	108	31.9	41%–50%	8	2.4
16–20 years	59	17.4	More than 50%	65	19.2
More than 20 years	54	15.9	Total	339	100
Total	339	100			

**Note:** 1 USD = 6.35 CNY.

The majority of the respondents, 215 (63.4%), were from small enterprises, while 124 (36.6%) were from medium enterprises. As for CBEC engagement, 34.8% of enterprises have never been involved in cross-border e-commerce, and 29.8% of companies’ CBEC sales accounted for less than 20 percent of total sales. Also, 24 companies involved in CBEC sales reached 21%–30%. Meanwhile, 6.8% and 2.4% of firms had CBEC sales from 31% to 40 % and 41% to 50 %, respectively. CBEC sales of 19.2% of enterprises accounted for more than half of their total sales.

### 4.2 Reliability and validity

In Table 3, the measures of validity and reliability for all constructs are shown. Following Hair et al. [88], Cronbach's alpha (CA), rho by Dijkstra-Hensele, and composite reliability were used to find the reliability of the study's latent constructs (CR). Table 3 has a list of the results. The values of Cronbach's alpha for every latent construct are higher than 0.70, and the lowest Cronbach's alpha value is 0.833 [97]. This showed that all of the things were true. Also, all of the study's constructs' rho values are well above the 0.70 threshold, with the lowest value being 0.836 [88]. This showed that the items in all constructs could be trusted. Also, the CR values were well over the 0.70 threshold, with the lowest being 0.888 [97]. This also proved that the items were real. For each construct, the average variance extracted (AVE) for each item must be higher than 0.50 to show enough convergent validity to support the idea of unidimensionality [88]. The results reflect that convergent validity exists. Last, the VIF values for every latent construct were all well below the standard value of 3.3, implying no multicollinearity [97].

**Table 3:** Reliability and validity.

Variables	No. Items	Mean	Standard Deviation	Cronbach's Alpha	DH rho	Composite Reliability	Average Variance Extracted	Variance Inflation Factors
CR	4	4.086	0.866	0.833	0.836	0.888	0.665	2.416
TK	4	4.232	0.837	0.848	0.850	0.898	0.688	2.788
FM	4	3.974	0.870	0.880	0.888	0.918	0.736	2.608
MP	4	3.761	1.092	0.929	0.931	0.949	0.825	1.655
MT	3	4.162	0.898	0.876	0.883	0.924	0.802	2.562
CM	4	4.311	0.823	0.877	0.876	0.916	0.734	2.475
MC	4	3.765	0.959	0.877	0.881	0.915	0.730	1.320
CP	4	4.101	0.816	0.859	0.871	0.905	0.705	1.118
SP	5	4.129	0.842	0.865	0.866	0.904	0.656	–

**Note:** CR: Collaborative Relationships, TK: Technical Knowledge and Abilities, FM: Flexible Manufacturing Competencies, MP: Modular Product Architecture, MT: Multi-talented Workforce, CM: Customer Relationship Management, MC: Mass Customization Capability, CP: Competitive Pressure, SP: Sustainable Performance.

**Fig. 2:**
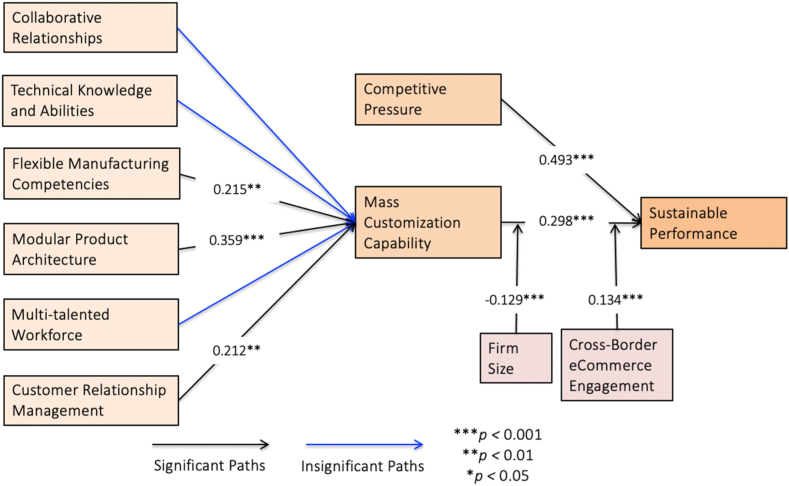
Measurement model.

The Fornell–Larcker Criterion, Hetro-trait and Mono-trait (HTMT) ratio, item loading, and cross-loading were also utilized in the present research to examine the discriminant validity (as presented inSupplementary Material S2. Validity Measurement). Comparing the factor loading and cross-loadings of the tested constructs was another way to check the discriminant validity of this study. Most of the time, loadings (Fig. 2) link an item to the latent construct to which it belongs [88]. Whereas, cross-loading is when an item adds to other latent constructs (seeSupplementary Material S2. Validity Measurement). The Fornell-Larcker criterion [101] was used to determine whether each study construct was discriminant. The square root of a construct's AVE is used to determine the Fornell-Larcker criterion. The correlation between the studies is lower than the square root of AVE for the construct. Next, the HTMT ratio of the study was used to test the discriminant validity [102]. All of the HTMT ratios were lower than the 0.900 limits, which showed that the study's latent variables had good discriminant validity [88].

### 4.3 Path analysis

To deduce how much of a given endogenous variable's variance can be accounted for by exogenous factors, examine its *r*^*2*^ value; this will indicate the sum of all the exogenous variables' effects on the endogenous variable. The rule of thumb states that *r*^*2*^ values of 0.75, 0.50, and 0.25 indicate substantial, moderate, and insignificant effects of independent variables on their corresponding endogenous counterparts accordingly [88]. Therefore, our results show moderate explanatory power (Table 4). By looking at how *r*^*2*^ changes when individual predictors are added or removed from the structural model, *f*^*2*^ shows the individual effects of an independent variable on a dependent variable [103]. The values of *f*^*2*^ that are greater than 0, 0.02, 0.15, and 0.35 show a small, average, or large effect of an independent factor on a dependent [104]. Based on that, our result presents a smaller effect for all the variables except competitive pressure, which shows extensive effects (Table 4).

**Table 4:** Hypothesis testing

Hypothesis		Beta	CI Min	CI Max	*t* values	*p* values	*f*^*2*^	*r*^*2*^	Decision
H_1a_	CR → MC	−0.026	−0.144	0.097	0.345	0.365	0.001	0.473	Reject
H_1b_	TK → MC	0.115	−0.025	0.243	1.413	0.079	0.009		Reject
H_1c_	FM → MC	0.215	0.079	0.341	2.697	0.004	0.034		Accept
H_1d_	MP → MC	0.359	0.254	0.463	5.669	0.000	0.148		Accept
H_1e_	MT → MC	−0.050	−0.186	0.077	0.628	0.265	0.002		Reject
H_1f_	CM → MC	0.212	0.105	0.343	2.887	0.002	0.034		Accept
H_2a_	MC → SP	0.298	0.227	0.364	7.098	0.000	0.132	0.492	Accept
H_2b_	CP → SP	0.493	0.411	0.572	10.066	0.000	0.428		Accept

**Note:** CR: Collaborative Relationships, TK: Technical Knowledge and Abilities, FM: Flexible Manufacturing Competencies, MP: Modular Product Architecture, MT: Multi-talented Workforce, CM: Customer Relationship Management, MC: Mass Customization Capability, CP: Competitive Pressure, SP: Sustainable Performance.

The empirical findings (Table 4 and Fig. 2) revealed that FM (beta value = 0.215, *t*-value = 2.697, *p*-value <0.001), MP (*β* = 0.359, *t* = 5.669, *p* < 0.001), CM (*β* = 0.212, *t* = 2.887, *p* < 0.001) have a significant positive effect on MC. In reverse, the influence of CR (*β* = −0.026, *t* = 0.345, *p* > 0.001), TK (*β* = 0.115, *t* = 1.143, *p* > 0.001), and MT (*β* = −0.050, *t* = 0.628, *p* > 0.001) is found insignificant on MC. The effects of MC (beta value = 0.298, *t* = 7.098, *p* < 0.001) and CP (*β* = 0.493, *t* = 10.066, *p* < 0.001) proved significant in the case of Chinese SMEs. Therefore, we accept H_1c_, H_1d,_ H_1f_, H_2a_, H_2b_ and reject H_1a,_ H_1b,_ H_1e._

### 4.4 Indirect and interaction effects

Table 5 shows that MC did not mediate the CR-SP relationship (*beta* value = −0.008, *t-value* = 0.346, *p*-value >0.05), TK and SP (*β* = 0.034, *t* = 1.399, *p* > 0.05), MT and SP (*β* = −0.015, *t* = −0.053, *p* > 0.05). Furthermore, a significant positive mediating effect of MC existed between FM and SP (*β* = 0.064, *t* = 2.460, *p* < 0.05), MP (*β* = 0.107, *t* = 4.631, *p* < 0.05), CM (*β* = 0.063, *t* = 2.708, *p* < 0.05) on SP.

**Table 5:** Indirect effect.

Association	Beta	CI Min	CI Max	*t* values	*p* values	*Decision*
CR → MC → SP	−0.008	−0.043	0.029	0.346	0.365	Reject
TK → MC → SP	0.034	−0.007	0.073	1.399	0.081	Reject
FM → MC → SP	0.064	0.022	0.107	2.460	0.007	Accept
MP → MC → SP	0.107	0.070	0.146	4.631	0.000	Accept
MT → MC → SP	−0.015	−0.053	0.024	0.634	0.263	Reject
CM → MC → SP	0.063	0.030	0.105	2.708	0.003	Accept

**Note:** CR: Collaborative Relationships, TK: Technical Knowledge and Abilities, FM: Flexible Manufacturing Competencies, MP: Modular Product Architecture, MT: Multi-talented Workforce, CM: Customer Relationship Management, MC: Mass Customization Capability, CP: Competitive Pressure, SP: Sustainable Performance.

**Fig. 3:**
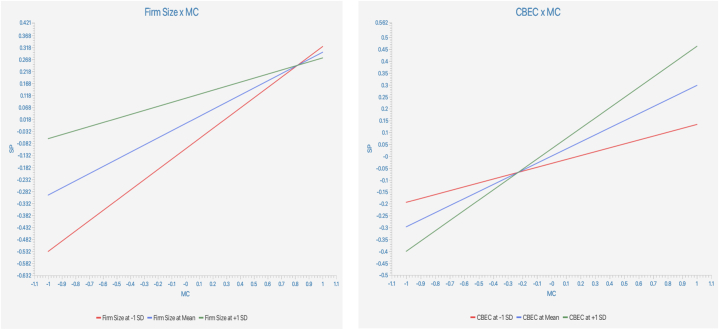
Moderating effect. **Note:** The two lines in the moderation analysis shows the relationship between SP: Sustainable Performance (y-axis) and MC: Mass Customization Capability (x-axis). The middle lines represent the moderating effect of Firm Size (right figure) and CABC: Cross-Border e-Commerce Engagement (left figure), where SD stands for standard deviation.

Regarding the moderating results, the study (Table 6 and Fig. 3) shows that the interaction of firm size between MC and SP is negatively significant (*beta value* = −0.129, *t*-value = 3.385, *p-value* < 0.05). Findings indicate that the positive effect of MC on SP is weaker (*f*^2^ = 0.025) for relatively larger firms than that for medium firms. Also, the analysis shows that CBEC plays a positive moderating on SP (*β* = 0.134, *t* = 2.910, *p* < 0.05). Findings specify that the positive influence of MC on SP is stronger (*f*^2^ = 0.031) for manufacturing SMEs with CBEC engagement.

**Table 6:** Moderating (interaction) effect.

Hypothesis		Beta	CI Min	CI Max	*t* values	*p* values	*f*^*2*^
H3a	Firm Size x MC ->SP	−0.129	0.050	0.200	3.385	0.000	0.025
H3b	CBEC x MC ->SP	0.134	−0.188	−0.062	2.910	0.002	0.031

**Note:** MC: Mass Customization Capability, SP: Sustainable Performance, CBEC: Cross-Border e-Commerce Engagement.

### 4.5 Necessary conditions analysis (NCA)

We added NCA to PLS-SEM to learn more about the connection between MC and its enablers. For a criterion to be deemed important, it must meet three requirements: it must have a theoretical basis, its effect size should be greater than zero, and its p-value must be small (<0.05). The NCA results are shown in Table 7 where FM (*d* = 0.089, *p-value* < 0.05), MP (*d* = 0.014, *p* < 0.05), CM (*d* = 0.246, *p* < 0.05) have statistically affected the MC of SMEs in china that confirmed previous results of this study. It is evident that CR (*d* = 0.180, *p* < 0.05), TK (*d* = 0.221, *p* < 0.05), and MT (*d* = 0.228, *p* < 0.05) also had a moderate and statistical effect on MC of Chinese SMEs, which was not identified in previous data analysis.

**Table 7:** NCA effect size.

Mass Customization Capability			Sustainable Performance		
Variables	Original effect size	Permutation p-value	Variables	Original effect size	Permutation p-value
CR	0.180	0.000	CP	0.265	0.000
TK	0.221	0.000	MC	0.024	0.008
FM	0.089	0.003	CBEC x MC	0.326	0.000
MP	0.014	0.027	Firm Size x MC	0.059	0.245
MT	0.228	0.000			
CM	0.246	0.000			

**Note:** CR: Collaborative Relationships, TK: Technical Knowledge and Abilities, FM: Flexible Manufacturing Competencies, MP: Modular Product Architecture, MT: Multi-talented Workforce, CM: Customer Relationship Management, MC: Mass Customization Capability, CP: Competitive Pressure, SP: Sustainable Performance, CBEC: Cross-Border e-Commerce Engagement.

Then, to offer more information, we conducted a “bottleneck analysis.” Table 7 shows the minimum values for the predictor constructs needed for each of the desired outcome variables in the first column. Table 8 shows that the level of comfort required to achieve a moderate level of MC (60–70%) is at least 2.36 percent. However, for MC to be high (100%), CR must reach a minimum of 15.044%. This means that a high level of MC will not happen if a predetermined minimum standard of CR (15.044%) is not reached. Similarly, TK (18.289%) and CM (17.699%) were also necessary for the advent of MC. A very low level of FM (9.44%), MP (10.029%), and MT (9.145%) were essential to attain the greatest level of MC.

**Table 8:** Bottleneck (percentages).

Mass Customization Capability	CR	TK	FM	MP	MT	CM
0%	NN	NN	NN	NN	1.475	NN
10%	NN	NN	NN	NN	1.475	NN
20%	NN	NN	NN	NN	1.475	NN
30%	NN	NN	NN	NN	1.475	NN
40%	NN	NN	NN	NN	1.475	NN
50%	NN	NN	NN	NN	1.475	NN
60%	2.36	3.835	NN	NN	1.475	3.835
70%	2.36	3.835	NN	NN	1.475	6.785
80%	4.72	3.835	NN	NN	1.475	7.375
90%	15.044	14.159	9.44	NN	9.145	17.699
100%	15.044	18.289	9.44	10.029	9.145	17.699

Sustainable Performance	CP	MC	Firm Size x MC	CBEC x MC
0%	NN	NN	NN	NN
10%	0.885	NN	NN	0.59
20%	1.18	NN	NN	0.59
30%	1.18	NN	NN	0.59
40%	1.18	NN	NN	0.59
50%	1.18	NN	NN	1.18
60%	1.18	NN	NN	1.18
70%	1.18	NN	NN	1.475
80%	1.18	NN	NN	1.475
90%	3.54	3.245	1.475	1.475
100%	7.67	6.195	2.065	1.475

**Note:** CR: Collaborative Relationships, TK: Technical Knowledge and Abilities, FM: Flexible Manufacturing Competencies, MP: Modular Product Architecture, MT: Multi-talented Workforce, CM: Customer Relationship Management, MC: Mass Customization Capability, CP: Competitive Pressure, SP: Sustainable Performance.

The necessary conditions for SP of Chinese manufacturing SMEs were depicted that MC (*d* = 0.024, *p* < 0.05) had a statistically but small effect on the SP. In contrast, CBEC x MC is necessary for SP, showing a large effect size (d = 0.326) [98], which is significant (*p* < 0.05). In addition, the NCA results are shown that CP significantly affected the SP of manufacturing enterprises in China. In particular, the essential conditions of the interaction (Firm Size x MC) were not met (*p* > 0.05). Hence, it was disregarded as relevant necessary conditions. The bottleneck analysis confirmed that 7.67 % of the CP, 6.195% of MC, and 1.475% of CBEC x MC were necessary for developing the SP of Chinese manufacturing SMEs.

## 5 Discussions

The study intends to find out the connection between MC, and its determinants, on SP. With its empirical investigation, the proposed model established a few relationships like MC and CP on the SP. The study also validated a few mediating relationships of MC between FM, MP, MT, and SP, except for a few others like CR, TK, and CM on MC. The moderating role of CBEC and firm size was also recognized in this investigation. The r^2^ value for mass customization was 47.3% of the total variation was explained by the predicts, while the total variation in SP was explained by MC and competitive pressure by 49.2%. Therefore, the SP model is a validated and well-fit model. The followings are the specific discussions of the hypothesis considered.

Past literature advocates that internal business strategies directly influence MC [2,3]. Thus, the study hypothesized that SP is predicted by the MC (H2a). The research outcomes are an attempt to guess the impact of MC, which are accountable for promoting the acceptance of SP in Chinese manufacturing SMEs. This indicates that enhanced MC promotes sustainable performance for SMEs. As the study's results show, FM significantly affects MC (H1c). This means that when advanced manufacturing technologies are combined with flexible MP, the consequence is the responsive skills essential to mass customization without too much cost, time, or process disruption. This result confirms the previous study of Ullah and Narain [2], who discovered the same relationships. The positive relationship between Flexible Manufacturing Competencies and Mass Customization Capability implies that organizations with greater competencies in flexible manufacturing are more likely to possess higher mass customization capabilities. Flexible manufacturing allows companies to adapt to varying customer demands, producing customized products efficiently. The findings support the idea that investments and improvements in flexible manufacturing processes positively contribute to a firm's ability to offer mass-customized products.

Also, the hypothesis (H1d) assumed that MP had a positive relationship with the MC, which is proved by this study. This corroborates the past study of Zhang et al. [59]. This signifies that the greater the FM and MP, the greater the propensity to form MC in the organizations. The result highlights the strategic importance of design principles that enable flexibility and customization. Companies incorporating modular product architectures are better positioned to respond efficiently to varying customer demands. This competitive advantage is crucial in markets where customers increasingly seek personalized products and services. Modular design facilitates the creation of customized products by allowing the interchangeability of components, reducing the complexity associated with customization. Organizations should view modular design not only as a technical aspect but also as a strategic lever for staying competitive in dynamic markets. Balancing modularity with integration and addressing operational challenges will be critical for realizing the full benefits of a modular approach. Similarly, CM also plays a statistically positive impact on MC, according to results in our study (H1e). This study confirms previous studies [2,63] in the Indian context. It indicates that customers must be a component of the value chain for mass customization to work. Mass customization cannot work without maintaining good links with customers. Hence, it is important to pay attention to CM practices, strategies, and technologies.

Whereas, CR, TK, and MT are supposed to have relationships with MC (H1a, H1b, and H1e). As opposed to the hypothesis, those three predictors of MC enablers gone wrong, i.e., found no links. The insignificant relationship between TK and MC is in reverse to the past research of Deshpande [21], who identified a significant relationship. This implies that greater collaborative relationships, technical knowledge, and abilities of employees (to solve floor problems and redesign equipment) and their multi-talented characteristics do not build the MC in the organization. The non-significant findings suggest that, within the studied context, factors related to collaborative relationships with external partners and technical knowledge may not be strong determinants of a firm's mass customization capability. It's important to consider the specific industry context in which these relationships are examined. Mass customization challenges and determinants may vary across industries. The non-significant results do not necessarily imply the absence of any relationship. It could be that the relationships are nuanced or contingent on other factors not considered in the study. To reach the company's long-term goals, a team of workers with different skills must work together. Cross-functional skills make it easier for workers to be creative and develop new ideas across the whole value chain. This lets firms reach a performance level that would not have been possible otherwise.

Similarly, the indirect relationships between MR, TK, MT, and SP via MC are supposed to be significant (H2a). However, it was found that MC has no indirect effect between collaborative relationships, TK and MT, and SP, as exhibited in this study. However, the NCA explains why these three enablers are not statistically significant to the MC because only when enterprises’ MC reaches a high level will these three enablers have an interaction effect between them. Due to this, mass customization requires organizations to work with their suppliers and customers. Setting up a strategic alliance with a supplier can offer the company access to complementary skills that can be relied on in times of trouble. Contrarily, an alliance with a customer provides the company with important information about the patterns of choices, preferences, and buying behavior that it can use to make decisions now and in the future. Moreover, TK makes product design and manufacturing more responsive, efficient, and accurate by letting designers manage the design and drawing of products and parts on the computer rather than on paper.

As expected, the study found a significant association between the firm external strategy, namely, the pressure of competition, and the SP of the manufacturing SEMs (H2b). This means that the contender orientation pushes the businesses to concentrate on continual improvement by managing the current processes, sequences, and schedules and making necessary changes. It also helps firms focus on creating unique and novel products and ways of doing things. Regarding the control variable, this study aimed to add insights by exploring whether CBEC can affect firm SP and the moderating role of MC and SP in SMEs (H3b). The results indicate that the successful development of CBEC can promote the MC of SMEs to improve their sustainable development in China. We found that firm size negatively connected with MC (H3a) for the other control variables. The possible reason is that the smaller the company, the more flexible technical, organizational, and workforce support can concentrate on MC.

## 6 Implications

### 6.1 Theoretical contribution

This study adds to the relevant knowledge generation in three ways. First, the study significantly contributes to the literature as there are few real-world studies about how different levels of MC enablers affect MC. The previous study confirmed the roles of MC factors such as individual-level strategies, information technology, and organizational structure. The factors that impact MC have been extensively covered in the relevant research [e.g., 32,33,48,105]. However, our study still brings new insight into MC research. The outcomes show that three out of six MC enablers, i.e., collaborative relationships, technical knowledge and abilities, and a multi-talented workforce have no impact on MC.

Second, further analysis using necessary condition analysis shows that the three enablers may affect MC only when the MC reaches a high level in manufacturing SEMs, enriching the MC enabler literature. The NCA incorporated into the study contributes significantly by not only validating existing findings but also uncovering previously undiscovered influences, providing practical guidance through bottleneck analysis, and extending the investigation to the link between MC and Sustainable Performance. These insights enhance the theoretical foundation of the study, offering valuable contributions for both academic research and strategic decision-making in the dynamic landscape of Chinese manufacturing SMEs.

Third, the incorporation of Competitive Pressure as a factor influencing Sustainable Performance extends theoretical models by recognizing the external dynamics that drive organizations toward sustainable practices. This finding suggests that organizations experiencing competitive pressures are more likely to translate their Mass Customization Capability into sustainable performance, emphasizing the importance of strategic alignment with market demands.

Fourth, this research adds to the literature by exploring the functions of MC as a mediator among its enablers and SP. Studies in the past have examined how MC acts as a link between its enablers and financial performance [106]. Our results show more indication of an obvious way for fully optimizing enablers to affect SP through MC. This makes the literature on MC thorough and richer and helps us understand how MC enablers affect performance. Finally, this study enriches the extant literature by presenting CBEC as a moderator for the association between MC and SP of SMEs. This research delivers a supplement to the literature opening up the moderating relationship between MC enablers, MC and SP, which enriches the MC and SP literature with the empirical test.

### 6.2 Practical implications

This study also offers several policy implications. First, this study looks at how and if six factors that make mass customization possible will affect MC. Interestingly, our necessary condition analysis findings reveal that collaborative relationships, technical knowledge, and the multi-talented workforce could affect MC only when mass customization reached a high level in manufacturing SMEs. It offers managers new ways to think about how to handle mass customization.

Second, our research shows that MC is linked with SP. This study suggests a way for managers to improve SP through MC. Management teams must pay attention to how vital mass customization is and how it affects performance. Besides, we found CR as a significant predictor of MC. Thus, the lesson for managers is that companies should always be on the lookout for more profitable ways to improve their relationships with customers. If employees do not work hard enough, firms might lose customers or get blown off course by changes. Owing to this, it is crucial to put more time and money into adopting the right CR tools and practices and building MC. To have a conquering mass customizer, managers must determine what their customers want and put that into the product specs while keeping costs and time as low as possible. To get “sticky” information, you need to be close to your customers. Thus, it is inevitable that businesses and customers will have a better experience and get better results if they use the right CR practices and automate their customer relationship processes.

Furthermore, organizations need to come up with and use the right strategies and practices, and they need to encourage employees to act in a way that helps them develop the cultural traits they want. These practices encompass developing flexible and modular teamwork of cross-functional staff that can be quickly rearranged based on needs, encouraging communication, the creation, and dissemination of information decentralizing decisions by giving employees some freedom, encouraging employees to come up with new ideas and sharing them without fear, making sure employees are safe and healthy, and restricting rules and bureaucratic procedures. Hence, organizations need to develop new policies and change the ones they already have. This could have a positive effect on how employees see their role in the success of the organization.

Third, we find that CBEC moderates the MC enablers and SP. Understanding how MC affects the MC enablers–SP links help managers to focus their hard work and efficient allocation of resources to fulfill the purpose. By doing so, managers can adequately understand how the effects of CBEC affect performance. MC improves how well a company does its job, which means that sustainable organizational practices will bring enormous benefits. CBEC companies need to perform better to integrate all of their customization capabilities into the way they run their businesses. To improve operational efficiency and, in turn, financial performance, collaboration and communication with suppliers and customers should be strengthened. For the SME's SPs, CBEC companies need to focus on internal capabilities and external competitors. Besides, technical knowledge and ability, such as information technology, improve sustained companies' performance through MC. Companies should transform into a data-driven system and its optimization. To ensure the successful integration of technology, companies should be ready to invest themselves or pool funds from external sources for the long-term and sustained performance of their competitors in the form of competitive advantage.

## 7 Conclusions, limitations and suggestions for future research

The findings of this study provide valuable insights into the relationships between various factors and their impact on MC and SP. Through a rigorous hypothesis testing process, we have examined the influence of CR, TK, FM, MP, MT, and CM on MC. Additionally, the study explored the effects of MC and CP on Sustainable Performance. The results reveal significant relationships between certain factors and both MC and SP. Specifically, Collaborative Relationships, Technical Knowledge and Abilities, Flexible Manufacturing Competencies, Modular Product Architecture, and Customer Relationship Management have been identified as key contributors to Mass Customization Capability. Conversely, Multi-talented Workforce exhibits a less significant relationship with MC. This nuanced understanding can inform human resource strategies, encouraging businesses to focus on targeted skill development and workforce optimization. Moreover, the study demonstrates that Mass Customization Capability, along with Competitive Pressure, positively influences Sustainable Performance. Businesses should prioritize building collaborative networks, fostering technical expertise, and developing agile manufacturing processes to strengthen their ability to deliver customized products efficiently. This insight becomes particularly relevant for businesses seeking to balance customization demands with the imperative of long-term environmental, social, and economic sustainability.

This study, like other studies, has some constraints, which could be tracked in future studies. First, while we have selected three significant regions, namely Guangdong, Shaanxi, and Zhejiang, as they are key manufacturing bases, it is important to note that these regions do not fully represent China as a whole. This is due to the diverse nature of the country's manufacturing sector, regional variations, industry specialization, size and scale, policy differences, technological disparities, and supply chain dynamics. Consequently, it restricts our capacity to investigate the causal connections between MC enablers, MC, and firm SP as they do not necessarily capture the diversity and variations that exist across the entire nation as far as cross-sectional data collection is concerned. Besides, as manufacturing landscapes, capabilities, and regulatory environments vary significantly across provinces and industries in China. Industry-specific nuances and diverse economic incentives, government support, and regulations are not fully captured by the chosen regions, limiting the broader applicability of findings to the entire country. Future studies can adopt longitudinal studies to manage such limitations. Longitudinal studies involve collecting data over an extended period, allowing researchers to observe changes and trends over time. This approach enables a more comprehensive understanding of how manufacturing capabilities evolve, how enablers contribute to these changes, and how they, in turn, affect a firm's strategic performance on a broader scale.

Second, in this study, six distinct MC enablers were employed to assess the relationship between these enablers and MC, with a subsequent analysis of the connection between MC and SP. It is worth noting that various other internal and external strategies can be independent variables to measure MC and SP. Advanced research could explore alternative mass customization strategies/enablers when examining the interplay between MC and SP in Chinese manufacturing SMEs. Third, CBEC plays a significant positive moderating between MC and SP. In the future, CBEC can also be predicted as an independent variable to measure a company's sustainable development. Finally, the present study revealed the moderation influence of CBEC, and FS on the relationship between MC and SP. However, additional moderators may also exist that affect the connection between MC and SP, and these should be considered in the study. To further understand the connections between MC and performance, future studies should examine a broader range of factors.

## Ethical approval

The human research ethics committee of Changzhi University have approved this study (Approval Number: CZ-2022-0082). This study has been performed in accordance with the Declaration of Helsinki.

## Informed consent

Written informed consent for participation was obtained from respondents who participated in the survey. No data was collected from anyone under 18 years old.

## Consent to publish

All authors approved the manuscript and give their consent for submission and publication.

## Funding

The study is supported via funding from (1) 10.13039/501100009103Department of Education of Shanxi Province (Grant no. 2021682) and (2) Shanxi Academy of Social Sciences (Grant no. YWQN202153).

## Availability of data and materials

Data included in article/supp. material/referenced in article.

## CRediT authorship contribution statement

**Guan Hui:** Writing – original draft, Methodology, Investigation, Conceptualization. **Abdullah Al Mamun:** Writing – review & editing, Methodology, Formal analysis, Conceptualization. **Mohammad Masukujjaman:** Writing – review & editing, Methodology, Formal analysis, Conceptualization. **Zafir Khan Mohamed Makhbul:** Writing – original draft, Methodology, Investigation, Conceptualization. **Mohd Helmi Ali:** Writing – original draft, Methodology, Investigation, Conceptualization.

## Declaration of competing interest

The authors declare that they have no known competing financial interests or personal relationships that could have appeared to influence the work reported in this paper.
