# Supplementary material for: The relationship between mass customization and sustainable performance: The role of firm size and global E-commerce
Source: Heliyon. 2024 Mar 11;10(6):e27726. doi: 10.1016/j.heliyon.2024.e27726 (PMC10950661; doi:10.1016/j.heliyon.2024.e27726)
Supplement: Multimedia component 1 [file mmc1.docx]

**Supplementary Material** **S1.** Survey instrument.

| Code | Item |
| --- | --- |
| CR1 | We are in frequent, close contact with our customers. |
| CR2 | Our customers are actively involved in our product design process. |
| CR3 | Our plant’s functions coordinate their activities. |
| CR4 | Management works well together on all important decisions. |
| TK1 | We allow customers to select appropriate features. |
| TK2 | We allow customers to dynamically generate the product code for new product configurations. |
| TK3 | We allow customers to master the production schedule. |
| TK4 | We receive and compare suppliers’ offers. |
| FM2 | Workers can perform many types of operations effectively. |
| FM3 | Cross-trained workers can perform a broad range of manufacturing tasks effectively in the organization. |
| FM4 | Material handling systems can move different part types through manufacturing facilities. |
| FM5 | The system has alternative routes in case machines break down. |
| MP1 | Our products are modularly designed, so they can be rapidly built by assembling modules. |
| MP2 | We have defined product platforms as a basis for future product variety and options. |
| MP3 | Our products are designed to use many common modules. |
| MP4 | When we make two products that differ only in one feature, we usually only need one different subassembly or component. |
| MT1 | Employees are cross-trained at this plant so that they can fill in for others if necessary. |
| MT2 | Employees receive training to perform multiple tasks. |
| MT4 | Many useful suggestions are implemented at our plant. |
| CM1 | We remain in contact with customers even after the deal is closed. |
| CM2 | We strive to acquire the knowledge and insight needed to deepen the relationships that matter. |
| CM5 | We make great efforts to re-establish relationships with valuable customers who have been inactive for some time. |
| CM6 | We provide customers bonus points or free gifts for their next purchases. |
| MC1 | We are highly capable of large-scale product customization. |
| MC2 | We can easily add significant product variety without increasing cost. |
| MC3 | Our setup costs, changing from one product to another, are very low. |
| MC5 | We can add product variety without sacrificing quality. |
| CP1 | Competition in our industry is cut-throat. |
| CP2 | There are many "promotion wars" in our industry. |
| CP3 | Anything that one competitor can offer, others can match easily. |
| CP4 | One hears of a new competitive move in our market frequently. |
| SP1 | Our organisation has reduced its overall energy consumption over the past five years. |
| SP2 | Our company's need for raw materials has decreased during the last five years. |
| SP3 | Our organisation has enhanced its adherence to environmental requirements during the past five years. |
| SP4 | There has been a decline in waste discharge during the past five years. |
| SP5 | Hazardous materials have been used less frequently in our organisation over the last five years. |

**Note:** CR: Collaborative Relationships, TK: Technical Knowledge and Abilities, FM: Flexible Manufacturing Competencies, MP: Modular Product Architecture, MT: Multi-talented Workforce, CM: Customer Relationship Management, MC: Mass Customization Capability, CP: Competitive Pressure, SP: Sustainable Performance
