# Supplementary material for: The relationship between mass customization and sustainable performance: The role of firm size and global E-commerce
Source: Heliyon. 2024 Mar 11;10(6):e27726. doi: 10.1016/j.heliyon.2024.e27726 (PMC10950661; doi:10.1016/j.heliyon.2024.e27726)
Supplement: Multimedia component 2 [file mmc2.docx]

**Supplementary Material** **S2.** Validity Measurement

|  | | | CR | | TK | FM | | MP | | MT | | CM | | MC | | CP | | SP |  |
| --- | --- | --- | --- | --- | --- | --- | --- | --- | --- | --- | --- | --- | --- | --- | --- | --- | --- | --- | --- |
| \| *Heterotrait-monotrait ratio (HTMT)* \| \| --- \| | | | | | | | | | |  | |  | |  | |  | |  |  |
| CR | | |  | |  |  | |  | |  | |  | |  | |  | |  |  |
| TK | | | 0.849 | |  |  | |  | |  | |  | |  | |  | |  |  |
| FM | | | 0.733 | | 0.731 |  | |  | |  | |  | |  | |  | |  |  |
| MP | | | 0.528 | | 0.515 | 0.655 | |  | |  | |  | |  | |  | |  |  |
| MT | | | 0.730 | | 0.728 | 0.801 | | 0.613 | |  | |  | |  | |  | |  |  |
| CM | | | 0.753 | | 0.830 | 0.681 | | 0.477 | | 0.757 | |  | |  | |  | |  |  |
| MC | | | 0.527 | | 0.590 | 0.643 | | 0.657 | | 0.553 | | 0.588 | |  | |  | |  |  |
| CP | | | 0.591 | | 0.614 | 0.484 | | 0.392 | | 0.574 | | 0.605 | | 0.358 | |  | |  |  |
| SP | | | 0.701 | | 0.675 | 0.683 | | 0.500 | | 0.771 | | 0.740 | | 0.546 | | 0.685 | |  |  |
| *Fornell-Larcker criterion* | | | | | | | | | |  | |  | |  | |  | |  |  |
| CR | | | 0.815 | |  |  | |  | |  | |  | |  | |  | |  |  |
| TK | | | 0.711 | | 0.829 |  | |  | |  | |  | |  | |  | |  |  |
| FM | | | 0.634 | | 0.636 | 0.858 | |  | |  | |  | |  | |  | |  |  |
| MP | | | 0.473 | | 0.459 | 0.596 | | 0.908 | |  | |  | |  | |  | |  |  |
| MT | | | 0.621 | | 0.623 | 0.704 | | 0.554 | | 0.896 | |  | |  | |  | |  |  |
| CM | | | 0.634 | | 0.715 | 0.604 | | 0.437 | | 0.657 | | 0.857 | |  | |  | |  |  |
| MC | | | 0.466 | | 0.519 | 0.579 | | 0.593 | | 0.495 | | 0.532 | | 0.854 | |  | |  |  |
| CP | | | 0.498 | | 0.530 | 0.429 | | 0.355 | | 0.501 | | 0.529 | | 0.320 | | 0.840 | |  |  |
| SP | | | 0.594 | | 0.578 | 0.602 | | 0.452 | | 0.669 | | 0.643 | | 0.484 | | 0.594 | | 0.810 |  |
| Loading and Cross Loading | | | | | | | | | | | | | | | | | | | |
| CR1 | 0.816 | 0.611 | | 0.480 | | | 0.284 | | 0.503 | | 0.626 | | 0.317 | | 0.489 | | 0.534 | |  |
| CR2 | 0.790 | 0.569 | | 0.477 | | | 0.401 | | 0.425 | | 0.472 | | 0.390 | | 0.404 | | 0.416 | |  |
| CR3 | 0.821 | 0.587 | | 0.609 | | | 0.445 | | 0.607 | | 0.511 | | 0.395 | | 0.399 | | 0.554 | |  |
| CR4 | 0.833 | 0.559 | | 0.495 | | | 0.392 | | 0.490 | | 0.482 | | 0.403 | | 0.351 | | 0.443 | |  |
| TK1 | 0.670 | 0.857 | | 0.500 | | | 0.376 | | 0.538 | | 0.553 | | 0.418 | | 0.456 | | 0.518 | |  |
| TK2 | 0.481 | 0.779 | | 0.464 | | | 0.375 | | 0.459 | | 0.432 | | 0.421 | | 0.359 | | 0.365 | |  |
| TK3 | 0.611 | 0.877 | | 0.577 | | | 0.396 | | 0.484 | | 0.662 | | 0.460 | | 0.433 | | 0.479 | |  |
| TK4 | 0.597 | 0.800 | | 0.564 | | | 0.375 | | 0.588 | | 0.719 | | 0.420 | | 0.510 | | 0.557 | |  |
| FM2 | 0.520 | 0.491 | | 0.882 | | | 0.469 | | 0.583 | | 0.475 | | 0.445 | | 0.338 | | 0.498 | |  |
| FM3 | 0.612 | 0.590 | | 0.910 | | | 0.513 | | 0.691 | | 0.617 | | 0.511 | | 0.410 | | 0.590 | |  |
| FM4 | 0.449 | 0.495 | | 0.778 | | | 0.511 | | 0.500 | | 0.361 | | 0.453 | | 0.329 | | 0.413 | |  |
| FM5 | 0.578 | 0.590 | | 0.857 | | | 0.544 | | 0.626 | | 0.591 | | 0.560 | | 0.385 | | 0.546 | |  |
| MP1 | 0.385 | 0.353 | | 0.479 | | | 0.898 | | 0.451 | | 0.351 | | 0.502 | | 0.260 | | 0.335 | |  |
| MP2 | 0.500 | 0.482 | | 0.590 | | | 0.912 | | 0.549 | | 0.461 | | 0.570 | | 0.378 | | 0.488 | |  |
| MP3 | 0.413 | 0.395 | | 0.539 | | | 0.917 | | 0.512 | | 0.379 | | 0.533 | | 0.312 | | 0.398 | |  |
| MP4 | 0.412 | 0.430 | | 0.550 | | | 0.905 | | 0.495 | | 0.391 | | 0.545 | | 0.331 | | 0.410 | |  |
| MT1 | 0.548 | 0.525 | | 0.608 | | | 0.528 | | 0.922 | | 0.552 | | 0.469 | | 0.416 | | 0.580 | |  |
| MT2 | 0.531 | 0.566 | | 0.623 | | | 0.484 | | 0.917 | | 0.584 | | 0.454 | | 0.429 | | 0.589 | |  |
| MT4 | 0.598 | 0.588 | | 0.668 | | | 0.477 | | 0.846 | | 0.637 | | 0.404 | | 0.511 | | 0.636 | |  |
| CM1 | 0.598 | 0.672 | | 0.569 | | | 0.369 | | 0.634 | | 0.889 | | 0.427 | | 0.535 | | 0.599 | |  |
| CM2 | 0.598 | 0.632 | | 0.553 | | | 0.318 | | 0.603 | | 0.899 | | 0.417 | | 0.490 | | 0.593 | |  |
| CM5 | 0.584 | 0.663 | | 0.569 | | | 0.384 | | 0.629 | | 0.882 | | 0.463 | | 0.443 | | 0.604 | |  |
| CM6 | 0.399 | 0.482 | | 0.383 | | | 0.408 | | 0.389 | | 0.747 | | 0.494 | | 0.349 | | 0.410 | |  |
| MC1 | 0.487 | 0.521 | | 0.596 | | | 0.467 | | 0.471 | | 0.539 | | 0.832 | | 0.260 | | 0.433 | |  |
| MC2 | 0.265 | 0.344 | | 0.385 | | | 0.536 | | 0.337 | | 0.363 | | 0.863 | | 0.216 | | 0.327 | |  |
| MC3 | 0.334 | 0.370 | | 0.445 | | | 0.525 | | 0.358 | | 0.354 | | 0.873 | | 0.195 | | 0.375 | |  |
| MC5 | 0.468 | 0.505 | | 0.521 | | | 0.504 | | 0.496 | | 0.526 | | 0.848 | | 0.395 | | 0.491 | |  |
| CP1 | 0.483 | 0.504 | | 0.403 | | | 0.296 | | 0.493 | | 0.493 | | 0.227 | | 0.895 | | 0.564 | |  |
| CP2 | 0.350 | 0.293 | | 0.255 | | | 0.259 | | 0.292 | | 0.305 | | 0.210 | | 0.750 | | 0.435 | |  |
| CP3 | 0.403 | 0.431 | | 0.310 | | | 0.237 | | 0.390 | | 0.415 | | 0.232 | | 0.849 | | 0.460 | |  |
| CP4 | 0.425 | 0.526 | | 0.450 | | | 0.390 | | 0.483 | | 0.540 | | 0.398 | | 0.858 | | 0.522 | |  |
| SP1 | 0.580 | 0.528 | | 0.561 | | | 0.396 | | 0.552 | | 0.471 | | 0.441 | | 0.449 | | 0.793 | |  |
| SP2 | 0.367 | 0.335 | | 0.313 | | | 0.441 | | 0.380 | | 0.305 | | 0.365 | | 0.479 | | 0.666 | |  |
| SP3 | 0.546 | 0.552 | | 0.606 | | | 0.320 | | 0.630 | | 0.663 | | 0.363 | | 0.523 | | 0.836 | |  |
| SP4 | 0.474 | 0.493 | | 0.530 | | | 0.369 | | 0.621 | | 0.611 | | 0.412 | | 0.467 | | 0.885 | |  |
| SP5 | 0.423 | 0.419 | | 0.409 | | | 0.293 | | 0.511 | | 0.542 | | 0.368 | | 0.476 | | 0.852 | |  |

**Note:** CR: Collaborative Relationships, TK: Technical Knowledge and Abilities, FM: Flexible Manufacturing Competencies, MP: Modular Product Architecture, MT: Multi-talented Workforce, CM: Customer Relationship Management, MC: Mass Customization Capability, CP: Competitive Pressure, SP: Sustainable Performance
